# Neighborhood Disadvantage and Access to Liver Transplant Referral for Severe Alcohol-Associated Hepatitis

**DOI:** 10.1001/jamanetworkopen.2026.2567

**Published:** 2026-03-19

**Authors:** Lauren D. Nephew, Thomas Cotter, Ashwani Singal, Juan Pablo Arab, Jaideep Behari, Qing Tang, Samer Gawrieh, Wanzhu Tu, Naga Chalasani

**Affiliations:** 1 Division of Gastroenterology and Hepatology, Department of Medicine, Indiana University School of Medicine, Indianapolis; 2 Division of Gastroenterology and Hepatology, Department of Medicine, The University of Texas Southwestern Medical Center, Dallas; 3 Division of Gastroenterology and Hepatology, Department of Medicine, University of Louisville School of Medicine, Louisville, Kentucky; 4 Division of Gastroenterology, Hepatology and Nutrition, Department of Medicine, Virginia Commonwealth University School of Medicine, Richmond; 5 Division of Gastroenterology, Hepatology and Nutrition, Department of Medicine, University of Pittsburgh School of Medicine, Pittsburgh, Pennsylvania; 6 Department of Biostatistics and Health Data Science, Indiana University School of Medicine, Indianapolis

## Abstract

**Importance:**

Severe alcohol-associated hepatitis (sAH) is a leading indication for liver transplant (LT). However, access to LT begins with referral, a step that may not have been previously characterized using prospective multicenter data with detailed measures of social determinants of health.

**Objective:**

To examine the association of clinical severity and neighborhood disadvantage with referral, wait-listing, transplant, and short-term mortality in patients hospitalized with sAH.

**Design, Setting, and Participants:**

This prospective multicenter cohort study was a secondary analysis of the Alcohol-Associated Hepatitis Network observational cohort. It included patients hospitalized with sAH (Model for End-Stage Liver Disease [MELD] >20) across 5 US transplant centers from May 6, 2019, to November 8, 2023. Clinical, demographic, and social determinants of health data, including the Area Deprivation Index (ADI), were collected prospectively.

**Exposures:**

Clinical severity (MELD score) and neighborhood-level disadvantage (ADI).

**Main Outcomes and Measures:**

The primary outcomes were referral for LT evaluation, wait-listing, and receipt of LT, and the secondary outcome was 180-day mortality. Logistic regression and generalized additive models were used to evaluate independent and interactive associations of MELD and ADI with outcomes.

**Results:**

The cohort included 325 patients (mean [SD] age, 44.8 [10.2] years; 197 males [60.6%]). The mean (SD) MELD score was 29.2 (7.6), and the mean (SD) ADI was 56.2 (24.4). Only 120 patients (36.9%) were referred for LT. In multivariable analysis, higher MELD scores were associated with lower referral odds (odds ratio, 1.13 [95% CI, 1.07-1.18]; *P* < .001). ADI was not associated with referral odds (odds ratio, 0.99 [95% CI, 0.97-1.00]; *P* = .06). Generalized additive model analyses demonstrated significant MELD × ADI interactions for referral (*P* for interaction = .01), wait-listing (*P* for interaction = .004), and mortality (*P* for interaction = .01). At a MELD score 20 to 30, referral probability was 40% to 60% among patients with an ADI less than 30 vs 20% among those with an ADI 30 or more. Among patients with a MELD score 20 to 30, mortality exceeded 20% in those with an ADI 60 or more compared with 10% to 20% in those with an ADI less than 20.

**Conclusions and Relevance:**

In this multicenter cohort study of severe alcohol-associated hepatitis, neighborhood disadvantage was associated with referral at intermediate MELD scores. These findings suggest that referral is a critical leverage point for interventions and that outreach and navigation strategies informed by social context may improve access to LT.

## Key Points

**Question:**

Is neighborhood disadvantage associated with referral for liver transplant in patients with severe alcohol-associated hepatitis?

**Findings:**

In this cohort study of 325 patients, neighborhood disadvantage was associated with referral at intermediate Model for End-Stage Liver Disease scores.

**Meaning:**

The findings of this study suggest that outreach strategies informed by social context may improve access to liver transplant and may be applicable across organ systems, as referral is a critical equity leverage point.

## Teaser

This cohort study examines the association of clinical severity and neighborhood disadvantage with liver transplant referral, wait-listing, receipt, and short-term mortality in patients hospitalized with severe alcohol-associated hepatitis.

## Introduction

Alcohol-associated liver disease (ALD) has emerged as the leading indication for liver transplant (LT) in the US, driven in part by a rise in alcohol-associated hepatitis (AH), a severe and often fatal manifestation of ALD.^1,2^ Recent national trends show that rates of AH have increased, particularly among younger adults and women.^3,4,5^ Despite improvements in short-term medical management, severe AH (sAH) continues to carry a high risk of early mortality, with estimates ranging from 20% to 40% within 6 months of diagnosis.^6^ For selected patients, early LT can offer a life-saving intervention; however, access remains inconsistent across populations.^7^

Access to LT for patients with ALD is associated with clinical and social determinants of health (SDOH) and potentially their interactions.^8^ Clinically, patients with ALD often face greater scrutiny during psychosocial evaluation, which may delay or preclude referral and listing. Social factors, including race and ethnicity, sex, and neighborhood disadvantage, are also associated with access to LT.^8,9^ In a national United Network for Organ Sharing analysis of patients with ALD, Black patients and women had significantly lower listing-to-death ratios compared with White patients and men, even after adjustment for regional disease burden.^10^ These findings indicate that disparities in access to LT for ALD persist and that early steps in the transplant care cascade are key points in which social disadvantage may exert its most influence.

Existing studies examining LT in AH are primarily retrospective and lack granular SDOH data.^11^ They rarely capture patient-level or area-level social factors that may influence access to care. Moreover, little is known about how these barriers operate across the LT care cascade, specifically at the referral step, in which the SDOH are likely to have the largest influence. Additionally, in 1 study, patients with underlying ALD presented with adverse SDOH beyond those seen in patients with other underlying chronic liver diseases, suggesting that social risk factors may exacerbate delays in referral or transplant eligibility in this population.^12^

To address these gaps, we conducted a secondary analysis of a prospective multicenter cohort of patients with sAH to examine the joint association of clinical severity and social disadvantage with key steps in the LT care cascade and mortality. This study, to our knowledge, provides the first prospectively collected multicenter data on referral patterns for sAH with linked neighborhood disadvantage. This study aims to identify where patients drop off the transplant pathway and inform targeted outreach and navigation to improve access to transplant.

## Methods

This cohort study is a secondary analysis of data from a prospective, multicenter observational cohort of patients hospitalized with well-characterized AH, conducted through the Alcoholic-Associated Hepatitis Network (AlcHepNet). The parent study, funded by the National Institute on Alcohol Abuse and Alcoholism (NIAAA), enrolled patients at 5 US transplant centers and was designed to investigate the biology, natural history, and treatment of AH. Additional details have been previously published.^13^ The study protocol was reviewed and approved centrally by the Western institutional review board at each participating center. Written informed consent was obtained from all participants or from their legally authorized representatives. The study followed the Strengthening the Reporting of Observational Studies in Epidemiology (STROBE) for cohort studies.

Patients were enrolled between May 6, 2019, and November 8, 2023, during an index hospitalization for AH. AH was diagnosed using the NIAAA criteria,^14^ which included being aged 18 years or older and having a history of heavy alcohol use (≥60 g/day for men or ≥40 g/day for women), a recent onset of jaundice, a serum bilirubin of 3 mg/dL or more (to convert bilirubin to micromoles per liter, multiply by 17.104), an aspartate aminotransferase (AST) of more than 50 U/L (to convert AST to microkatals per liter, multiply by 0.0167), and an AST to alanine aminotransferase (to convert alanine aminotransferase to microkatals per liter, multiply by 0.0167) ratio of more than 1.5. Liver biopsy was performed when clinically indicated but was not required for enrollment. Patients with chronic viral hepatitis, autoimmune hepatitis, drug-induced liver injury, or other confounding causes of liver disease were excluded. For this analysis, only patients with sAH, defined as a Model for End-Stage Liver Disease (MELD) score more than 20, which ranges from 6 to 40, with higher scores indicating severe liver disease, were included, given the study’s focus on the LT care cascade.

### Clinical, Demographic, and SDOH Variables

Data collected at baseline and during follow-up of the observational study were complemented by a structured medical record review. Clinical variables included the MELD score and its components, liver chemistries, kidney function, coagulation parameters, and hematologic indices. Alcohol consumption data and corticosteroid use during the index hospitalization were also collected.

Demographic and SDOH variables included age, sex, race, ethnicity, marital status, educational level attainment (categorized as high school or less vs education beyond high school), employment status, and insurance type (classified as private, Medicaid, Medicare, uninsured, or other). Race categories included American Indian or Alaska Native, Asian, Black or African American, White, multiple races (American Indian or Alaska Native and White), and unknown; ethnicity categories included Hispanic or Latino and non-Hispanic or non-Latino. Race and ethnicity were ascertained by self-report; these data were collected because they were hypothesized to be associated with referral.

Patients were also asked whether they had children living at home. We assessed neighborhood-level social disadvantage using the Area Deprivation Index (ADI), a validated composite measure of socioeconomic disadvantage based on 17 census-derived indicators including income, educational level, employment, and housing quality (scores range from 1 to 100, with higher scores indicating greater disadvantage).^15^ ADI scores were linked to patients’ residential 9-digit zip codes at enrollment.

### Outcomes

The primary outcomes of interest were sequential steps in the LT care cascade: referral for transplant, wait-listing for transplant, and receipt of LT. The secondary outcome was 180-day all-cause mortality. Outcomes were assessed through longitudinal review of the electronic health record and study follow-up documentation.

### Statistical Analysis

Demographic and clinical characteristics of the study participants were described by referral, wait-listing, and transplant status. Categorical variables were summarized as frequency (percentage) and compared using χ^2^ tests; continuous variables were summarized with mean (SD) and compared using 2-sample *t* tests. We examined the distributions of the continuous variables. For variables with skewed distributions, we reported values of the medians and IQRs; comparisons were made using the nonparametric Wilcoxon rank sum test. We conducted separate logistic regression analyses to assess the association between participant characteristics and referral, wait-listing, transplant, and 180-day survival. Patient characteristics included age, sex, race, ethnicity, educational level, insurance status, marital status, living with children, MELD score, ADI, and study site. We screened the patient characteristics using bivariate regression models, including 1 characteristic at a time; variables with *P* values less than .1 were considered for possible inclusion. The final logistic regression models were assessed based on the existing literature and variable screening results. We then fitted generalized additive models (GAMs) with a logit link function for the noted outcomes, using the same set of patient characteristic variables. A GAM is a newer semiparametric regression technique, often used for discovering nonlinear and interactive effects of independent variables on the response variable.^16^ A GAM with a logit link function for binary outcomes is similar to logistic regression but is able to accommodate nonlinear effects through included spline terms. In this analysis, we used a GAM to assess the concurrent association of AH severity (MELD) and access disadvantage. Here, the association of MELD and ADI with LT access was modeled as a bivariate smooth function. The simultaneous associations of MELD and ADI estimated from GAMs were presented in colored contour plots, in which warmer colors (yellow shades) indicate higher probabilities of having the outcome, and colder colors (blue shades) indicate lower probabilities.^17,18^ Covariates included in the GAMs mirrored those used in the multivariable logistic regression models; variables with *P* < .10 did not meet screening criteria.

In the multivariable logistic and GAM models, insurance was included as a binary covariate (private insurance [reference level] vs others). Because Medicaid coverage of transplant is not uniform across sites, and multiple insurance types influence willingness to refer, we conducted a sensitivity analysis to assess the robustness of the analytical results. Specifically, we used a 5-level categorical variable for insurance (private insurance, Medicaid, Medicare, uninsured, and other). This was done at the referral step, in which the sample size supported stable estimation across the multiple categories. To evaluate potential site-level heterogeneity, we tested an interaction between insurance and site. Finally, we refit the GAM for the referral outcome.

Analyses were performed with SAS software, version 9.3 (SAS Institute Inc) and package mgcv in R, version 1.9-4 (R Project for Statistical Computing).^18^ Two-sided *P* < .05 was considered statistically significant.

## Results

### Patient Characteristics

A total of 325 patients with sAH and a MELD score more than 20 (mean [SD] age, 44.8 [10.2] years; 128 females [39.4%] and 197 males [60.6%]) were included in the study. Most patients identified as White (280 [86.2%]); 4 (1.2%) were American Indian or Alaska Native, 2 (0.6%) were Asian, 30 (9.2%) were Black or African American, 38 (12.0%) were Hispanic or Latino, 279 (88.0%) were non-Hispanic or non-Latino, 2 (0.6%) were multiple races, and 7 (2.2%) were of unknown race. Of the total patients, 145 (45.5%) had a high school education or less; the mean (SD) MELD score was 29.2 (7.6). Key MELD components included a median (IQR) serum bilirubin of 19.7 (10.3-28.6) mg/dL, a mean (SD) international normalized ratio of 2.1 (0.6), and a median (IQR) creatinine of 1.0 (0.7-2.1) mg/dL (to convert creatinine to micromoles per liter, multiply by 88.4). The mean (SD) albumin was 2.8 (0.6) g/dL (to convert albumin to grams per liter, multiply by 10). Socioeconomic burden was high, with a mean (SD) ADI of 56.2 (24.4), and 79 patients (24.4%) were uninsured (Table 1).

**Table 1.** Demographic and Baseline Characteristics of Referral Status for Liver Transplant for Patients With Severe Acute Alcohol-Associated Hepatitis

Variable	Overall (N = 325)	Without referral (n = 205)	With referral (n = 120)	*P* value^a^	SMD
Age, mean (SD), y	44.8 (10.2)	45.5 (10.4)	43.5 (9.7)	.10	−0.20
Sex, No. (%)					
Female	128 (39.4)	83 (40.5)	45 (37.5)	.59	NA
Male	197 (60.6)	122 (59.5)	75 (62.5)		
Race, No. (%)					
American Indian or Alaska Native	4 (1.2)	1 (0.5)	3 (2.5)	.12	NA
Asian	2 (0.6)	1 (0.5)	1 (0.8)		
Black or African American	30 (9.2)	24 (11.7)	6 (5.0)		
White	280 (86.2)	172 (83.9)	108 (90.0)		
Multiple races^b^	2 (0.6)	2 (1.0)	NA		
Unknown	7 (2.2)	5 (2.4)	2 (1.7)		
Ethnicity, No. (%)					
Hispanic or Latino	38 (12.0)	31 (15.5)	7 (6.0)	.01	NA
Non-Hispanic or non-Latino	279 (88.0)	169 (84.5)	110 (94.0)		
BMI, mean (SD)	30.3 (7.4)	30.4 (7.4)	30.2 (7.4)	.85	−0.02
MELD score, mean (SD)^c^	29.2 (7.6)	27.4 (6.9)	32.3 (7.7)	<.001	0.68
Received corticosteroids, No. (%)					
No	223 (68.6)	141 (68.8)	82 (68.3)	.93	NA
Yes	102 (31.4)	64 (31.2)	38 (31.7)		
Albumin, mean (SD), g/dL	2.8 (0.6)	2.7 (0.5)	3.0 (0.7)	<.001	0.38
Total serum bilirubin, median (IQR), mg/dL	19.7 (10.3-28.6)	17.3 (9.1-26.0)	23.7 (17.1-32.1)	<.001	0.50
Creatinine, median (IQR), mg/dL	1.0 (0.7-2.1)	0.9 (0.6-1.4)	1.5 (0.8-3.2)	<.001	0.53
INR, mean (SD)	2.1 (0.6)	2.1 (0.6)	2.2 (0.6)	.01	0.29
Prothrombin time, mean (SD), s	23.9 (6.4)	23.4 (6.2)	24.8 (6.8)	.06	0.21
AST, median (IQR), U/L	110.0 (80.0-158.0)	117.0 (82.0-161.0)	105.5 (75.0-145.0)	.12	−0.18
ALT, median (IQR), U/L	39.0 (26.0-60.5)	39.0 (27.0-58.0)	39.0 (24.0-63.0)	.70	−0.04
Alkaline phosphatase, median (IQR), U/L	157.0 (119.0-220.0)	168.0 (117.0-229.0)	149.5 (120.0-199.0)	.17	−0.16
Fasting glucose, mean (SD), mg/dL	110.8 (30.2)	110.0 (29.5)	113.3 (33.2)	.75	0.10
Total protein, mean (SD), g/dL	5.8 (0.9)	5.8 (0.9)	5.7 (0.9)	.11	−0.19
Hemoglobin, mean (SD), g/dL	9.3 (1.8)	9.4 (1.8)	9.1 (1.8)	.15	−0.16
WBC count, median (IQR), ×10^3^/L	11.9 (8.2-17.1)	11.2 (7.8-16.4)	13.4 (9.2-19.3)	.01	0.30
Platelet count, median (IQR), ×10^3^/L	120.0 (78.0-184.5)	128.0 (81.0-194.0)	112.0 (75.0-169.0)	.10	−0.20
MCV, mean (SD), μm^3^	100.6 (8.3)	101.2 (8.8)	99.6 (7.3)	.09	−0.20
Educational level, No. (%)					
≤High school	145 (45.5)	106 (53.0)	39 (32.8)	<.001	NA
College graduate	174 (54.5)	94 (47.0)	80 (67.2)		
Insurance type, No. (%)					
Private	106 (32.7)	52 (25.4)	54 (45.4)	<.001	NA
Medicaid	79 (24.4)	44 (21.5)	35 (29.4)		
Medicare	42 (13.0)	25 (12.2)	17 (14.3)		
None	79 (24.4)	67 (32.7)	12 (10.1)		
Other	18 (5.6)	17 (8.3)	1 (0.8)		
Area Deprivation Index, mean (SD)^d^	56.2 (24.4)	60.3 (23.2)	49.1 (25.1)	<.001	−0.46
Employed, No. (%)					
No	195 (61.1)	129 (64.8)	66 (55.0)	.08	NA
Yes	124 (38.9)	70 (35.2)	54 (45.0)		
Marital status, No. (%)					
Divorced, separated, widowed, or single	186 (57.6)	123 (60.3)	63 (52.9)	.20	NA
Married or living with partner	137 (42.4)	81 (39.7)	56 (47.1)		
Spouse living at home, No. (%)					
No	7 (6.0)	6 (9.2)	1 (1.9)	.13	NA
Yes	110 (94.0)	59 (90.8)	51 (98.1)		
Children living at home, No. (%)					
Does not have children	86 (26.7)	49 (24.3)	37 (30.8)	.03	NA
No	121 (37.6)	87 (43.1)	34 (28.3)		
Yes	115 (35.7)	66 (32.7)	49 (40.8)		
Age began drinking alcohol, mean (SD), y	19.1 (6.2)	19.1 (6.5)	19.1 (5.6)	.98	−0.002
Standard alcoholic drinks, mean (SD) per d	11.0 (8.1)	10.9 (8.3)	11.3 (7.7)	.64	0.06

Abbreviations: ALT, alanine aminotransferase; AST, aspartate aminotransferase; BMI, body mass index (calculated as weight in kilograms divided by height in meters squared); INR, international normalized ratio; MCV, mean corpuscular volume; MELD, Model for End-Stage Liver Disease; NA, not applicable; SMD, standardized mean difference; WBC, white blood cell.

SI conversions: To convert albumin to grams per liter, multiply by 10; total bilirubin to micromoles per liter, multiply by 17.104; creatinine to micromoles per liter, multiply by 88.4; prothrombin time to seconds, multiply by 1.0; ALT, AST, and alkaline phosphatase to microkatals per liter, multiply by 0.0167; fasting glucose to millimoles per liter, multiply by 0.0555; total protein and hemoglobin to grams per liter, multiply by 10.0; white blood cell count to ×10^9^ per liter, multiply by 0.001; platelet count to ×10^9^ per liter, multiply by 1.0; MCV to femtoliters, multiply by 1.0.

^a^ Categorical variables were summarized as No. (%) and compared using χ^2^ tests; continuous variables were summarized with mean (SD) using 2-sample *t* tests; and comparisons were made using nonparametric Wilcoxon rank sum test.

^b^ Includes American Indian or Alaska Native and White.

^c^ Scores range from 6 to 40, with higher scores indicating severe liver disease.

^d^ Scores range from 1 to 100, with higher scores indicating greater disadvantage.

In total, 120 patients (36.9%) were referred for LT. Of those referred, 58 (48.3%) were wait-listed, and 32 (55.2%) of wait-listed patients received LT. Overall, 32 of 325 (9.8%) of the full cohort underwent LT. At 180 days, 83 patients (25.5%) had died, and 242 (74.5%) were alive (eTable 1 in Supplement 1).

Compared with those who were not referred, patients who were referred for LT evaluation had significantly higher mean (SD) MELD scores (32.3 [7.7] vs 27.4 [6.9]; *P* < .001) and lower mean (SD) ADI values (49.1 [25.1] vs 60.3 [23.2]; *P* < .001). Referred patients vs patients who were not referred were more likely to have private insurance (45.4% vs 25.4%; *P* < .001) and higher levels of education (67.2% with education beyond high school vs 47.0% without education beyond high school; *P* < .001) and were less likely to be Hispanic or Latino (6.0% vs 15.5%; *P* = .01) (Table 1).

### Factors Associated With Referral for Transplant Evaluation

Of the 325 patients hospitalized with sAH, 120 (36.9%) were referred for LT evaluation. In multivariable logistic regression, adjusting for age, sex, race, ethnicity, educational level, insurance, marital status, living with children, MELD score, ADI, and site of care, a higher MELD score was associated with increased odds of referral (odds ratio [OR], 1.13 [95% CI, 1.07-1.18]; *P* < .001) (Table 2). Private insurance (OR, 1.91 [95% CI, 0.98-3.74]; *P* = .06) and higher educational level (OR, 1.87 [95% CI, 0.98-3.55]; *P* = .06) were not associated with referral. A higher ADI was not statistically significantly associated with lower referral odds (OR, 0.99 [95% CI, 0.97-1.00]; *P* = .06). On multivariable analysis, compared with site 1, the odds of patient referral for LT were significantly higher at site 2 (OR, 4.08 [95% CI, 1.04-16.03]; *P* = .04), site 3 (OR, 3.90 [95% CI, 1.12-13.62]; *P* = .03), and site 5 (OR, 4.80 [95% CI, 1.52-15.19]; *P* = .008) (Table 2). There was significant center-level variability in referral patterns, with patients at some sites having 4 to 5 times higher odds of referral (eg, site 5: OR, 5.62 [95% CI, 1.68-18.83]).

**Table 2.** Multivariable Logistic Regression Analyses at Each Step in the Liver Transplant Cascade^a^

Factor	Referral (n = 299)		Wait-listing (n = 114)		Transplant (n = 66)		180-d Mortality (n = 311)	
	OR (95% CI)	*P* value	OR (95% CI)	*P* value	OR (95% CI)	*P* value	OR (95% CI)	*P* value
Age	1.00 (0.97-1.03)	.89	1.00 (0.95-1.05)	.97	1.00 (0.93-1.08)	.94	1.03 (1.00-1.06)	.03
Sex								
Female	1 [Reference]	NA	1 [Reference]	NA	1 [Reference]	NA	1 [Reference]	NA
Male	1.34 (0.72-2.48)	.35	0.40 (0.14-1.14)	.09	1.25 (0.25-6.28)	.79	0.80 (0.44-1.45)	.46
Race								
Black or African American	0.71 (0.24-2.11)	.54	0.26 (0.03-2.71)	.26	<.001 (0->999.99)	.98	1.56 (0.63-3.87)	.34
White	1 [Reference]	NA	1 [Reference]	NA	1 [Reference]	NA	1 [Reference]	NA
Other or unknown^b^	3.09 (0.85-11.26)	.09	0.31 (0.04-2.41)	.26	0.48 (0.01-17.47)	.69	0.53 (0.11-2.65)	.44
Ethnicity								
Hispanic or Latino	0.56 (0.17-1.84)	.34	NR	NR	NR	NR	NR	NR
Non-Hispanic or non-Latino	1 [Reference]	NA	1 [Reference]	NR	1 [Reference]	NR	1 [Reference]	NR
MELD score (≤40)^c^	1.13 (1.07-1.18)	<.001	1.10 (1.02-1.18)	.02	1.24 (1.07-1.44)	.004	NR	NR
MELD score^c^	NR	NR	NR	NR	NR	NR	1.07 (1.03-1.12)	<.001
Albumin	1.21 (0.74-1.97)	.45	NR	NR	NR	NR	NR	NR
Educational level								
Trade school, college, or graduate program	1.87 (0.98-3.55)	.06	NR	NR	NR	NR	NR	NR
≤High school	1 [Reference]	NA	NR	NR	NR	NR	NR	NR
Insurance								
Private	1.91 (0.98-3.74)	.06	NR	NR	NR	NR	NR	NR
Other	1 [Reference]	NA	NR	NR	NR	NR	NR	NR
Area Deprivation Index^d^	0.99 (0.97-1.00)	.06	0.98 (0.96-1.00)	.02	0.99 (0.96-1.02)	.38	1.01 (1.00-1.02)	.18
Marital status								
Married or living with significant other	0.91 (0.48-1.73)	.77	2.04 (0.81-5.13)	.13	1.83 (0.36-9.25)	.47	0.78 (0.44-1.39)	.40
Divorced, separated, widowed, or single	1 [Reference]	NA	1 [Reference]	NA	1 [Reference]	NA	1 [Reference]	NA
Children living at home								
No	0.82 (0.37-1.84)	.64	NR	NR	NR	NR	NR	NR
Yes	1.44 (0.68-3.05)	.35	NR	NR	NR	NR	NR	NR
Do not have children	1 [Reference]	NA	1 [Reference]	NA	1 [Reference]	NA	1 [Reference]	NA
Employed	1.43 (0.77-2.66)	.26	1.92 (0.72-5.10)	.19	4.80 (0.84-27.40)	.08	0.72 (0.39-1.33)	.30
Site								
2	4.08 (1.04-16.03)	.04	0.63 (0.07-5.95)	.69	NR	NR	0.63 (0.23-1.76)	.38
3	3.90 (1.12-13.62)	.03	1.35 (0.16-11.57)	.78	NR	NR	0.36 (0.11-1.12)	.08
4	2.67 (0.74-9.56)	.13	1.10 (0.11-11.56)	.93	NR	NR	0.71 (0.27-1.87)	.49
5	4.80 (1.52-15.19)	.008	1.64 (0.22-12.22)	.63	NR	NR	0.52 (0.21-1.26)	.15

Abbreviations: MELD, Model for End-Stage Liver Disease; NA, not applicable; NR, not reported; OR, odds ratio.

^a^ Multivariable logistic regression models were fit based on clinical judgment and the variables, with *P* < .10 from univariable regression models.

^b^ Collapsed in the model analysis to include American Indian or Alaska Native, Asian, multiple races, and unknown races.

^c^ Scores range from 6 to 40, with higher scores indicating severe liver disease.

^d^ Scores range from 1 to 100, with higher scores indicating greater disadvantage.

**Figure 1.**
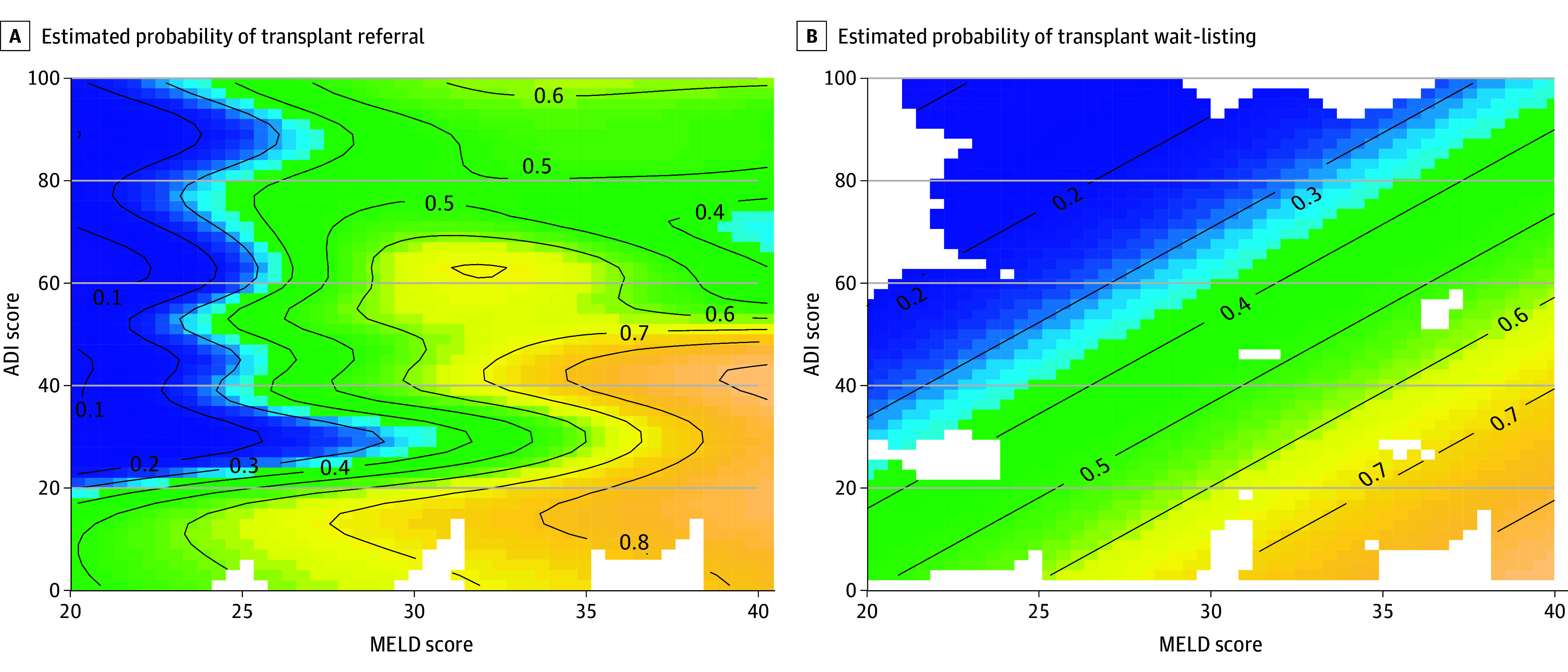
Association of the Area Deprivation Index (ADI) Score and the Model for End-Stage Liver Disease (MELD) Score With Transplant Referral and Wait-Listing Estimated probability of transplant referral (A) and wait-listing (B) as a smooth function of the MELD score and neighborhood disadvantage (ADI). Patients with a higher ADI had significantly lower estimated referral probabilities at lower MELD scores (blue), with probabilities rising at higher MELD scores (yellow), and higher MELD and lower ADI values were associated with increased estimated wait-listing probabilities.

In the GAM analysis, the interaction between MELD and ADI was associated with referral probability (*P* for interaction = .01) (Table 3). The estimated probability of referral as a smooth function of MELD and ADI, with warmer colors indicating higher referral probabilities, is shown in Figure 1A. At lower MELD scores (20-30), the probability of referral varied sharply by ADI, with a steep gradient from approximately 50% among patients living in the least-deprived areas (ADI <30) to less than 20% for those in more deprived areas (ADI ≥30). This trend flattened at higher MELD scores (>30), in which referral probability exceeded 50% across nearly all ADI levels. Referral probabilities were highest among patients with high MELD and low ADI (eTable 2 in Supplement 1).

**Table 3.** Generalized Additive Models Evaluating MELD and ADI Interactions Across the Liver Transplant Cascade

Covariate	Referral (n = 299)		Wait-listing (n = 114)		Transplant (n = 66)		180-d Mortality (n = 311)	
	OR (95% CI)	*P* value	OR (95% CI)	*P* value	OR (95% CI)	*P* value	OR (95% CI)	*P* value
Age	1.00 (0.96-1.03)	.89	1.00 (0.95-1.05)	.97	1.01 (0.93-1.11)	.76	1.03 (1.00-1.06)	.04
Sex								
Female	1 [Reference]	NA	1 [Reference]	NA	1 [Reference]	NA	1 [Reference]	NA
Male	1.35 (0.68-2.68)	.40	0.40 (0.14-1.14)	.09	0.60 (0.07-4.81)	.63	0.79 (0.41-1.50)	.47
Race								
Black or African American	0.67 (0.22-2.09)	.49	0.27 (0.03-2.71)	.26	<.001 (0->999.99)	>.99	1.91 (0.71-5.13)	.20
White	1 [Reference]	NA	1 [Reference]	NA	1 [Reference]	NA	1 [Reference]	NA
Other or unknown^a^	3.02 (0.77-11.87)	.11	0.31 (0.04-2.41)	.26	0.41 (0.01-26.82)	.68	0.41 (0.07-2.30)	.31
Ethnicity^b^								
Hispanic or Latino	0.81 (0.23-2.88)	.74	NR	NR	NR	NR	NR	NR
Albumin	1.31 (0.79-2.18)	.30	NR	NR	NR	NR	NR	NR
Educational level								
Trade school, college, or graduate program	2.31 (1.16-4.60)	.02	NR	NR	NR	NR	NR	NR
≤High school	1 [Reference]	NA	NR	NR	NR	NR	NR	NR
Insurance								
Private	2.03 (1.00-4.14)	.05	NR	NR	NR	NR	NR	NR
Other	1 [Reference]	NA	NR	NR	NR	NR	NR	NR
Marital status								
Married or living with significant other	0.82 (0.41-1.64)	.57	2.04 (0.81-5.13)	.13	1.73 (0.25-12.07)	.58	0.90 (0.48-1.67)	.73
Divorced, separated, widowed, or single	1 [Reference]	NA	1 [Reference]	NA	1 [Reference]	NA	1 [Reference]	NA
Children living at home								
No	1.08 (0.44-2.63)	.87	NR	NR	NR	NR	NR	NR
Yes	1.63 (0.71-3.77)	.25	NR	NR	NR	NR	NR	NR
Does not have children	1 [Reference]	NA	NR	NR	NR	NR	NR	NR
Employed	1.55 (0.79-3.02)	.20	1.92 (0.72-5.10)	.19	10.65 (1.25-90.81)	.03	0.69 (0.36-1.32)	.26
Site								
2	5.17 (1.25-21.30)	.02	0.63 (0.07-5.95)	.69	NR	NR	0.56 (0.18-1.73)	.31
3	4.59 (1.21-17.49)	.03	1.35 (0.16-11.57)	.78	NR	NR	0.32 (0.09-1.09)	.07
4	2.50 (0.65-9.63)	.18	1.10 (0.11-11.56)	.93	NR	NR	0.57 (0.20-1.66)	.30
5	5.62 (1.68-18.83)	.005	1.64 (0.22-12.22)	.63	NR	NR	0.44 (0.17-1.17)	.10
S (ADI and MELD ≤40)^c^	NA	.01	NA	.004	NA	.38	NR	NR
S (ADI and MELD)^c^	NR	NR	NR	NR	NR	NR	NA	.01

Abbreviations: ADI, Area Deprivation Index; MELD, Model for End-Stage Liver Disease; NA, not applicable; NR, not reported; OR, odds ratio; S, smooth, nonlinear terms in Generalized Additive Models.

^a^ Collapsed in the model analysis to include American Indian or Alaska Native, Asian, multiple races, and unknown races.

^b^ Data for non-Hispanic or non-Latino were not reported because results were not significant on univariable analysis.

^c^ ADI scores range from 1 to 100, with higher scores indicating greater disadvantage; MELD scores range from 6 to 40, with higher scores indicating severe liver disease.

In the sensitivity analysis, there was no evidence that the insurance and referral association varied by site (eTable 3 in Supplement 1). In the GAM, results were consistent: uninsured patients had lower odds of referral vs privately insured (OR, 0.15 [95% CI, 0.05-0.40]; *P* < .001), and the MELD and the ADI smooth term remained significant (eTable 4 in Supplement 1).

### Factors Associated With Wait-Listing

Among the 120 referred patients, 58 (48.3%) were wait-listed. In multivariable analysis, the MELD score was associated with wait-listing (OR, 1.10 [95% CI, 1.02-1.18]; *P* = .02), as was ADI (OR, 0.98 [95% CI, 0.96-1.00]; *P* = .02) (Table 2). The site of care and other clinical or social variables were not associated with wait-listing.

The GAM analysis revealed a significant interaction between MELD and ADI (*P *for interaction = .004) (Table 3). Wait-listing probability was highest among patients with a MELD score more than 30 and an ADI less than 40, for which it exceeded 60% (Figure 1B). In contrast, patients with similar MELD scores but a higher ADI (≥40) had significantly lower probabilities (approximately 20%-30%). For those with MELD scores of 20 to 30, wait-listing probability declined from approximately 40% at low ADI (<30) to approximately 25% at higher ADI (≥40) levels.

### Factors Associated With LT

Of the 58 wait-listed patients, 32 (55.2%) received LT. In multivariable analysis, a MELD score was associated with LT (OR, 1.24 [95% CI, 1.07-1.44]; *P* = .004) (Table 2). Employment (OR, 4.80 [95% CI, 0.84-27.40]; *P* = .08) and ADI (OR, 0.99 [95% CI, 0.96-1.02]; *P* = .38) were not associated with LT receipt.

**Figure 2.**
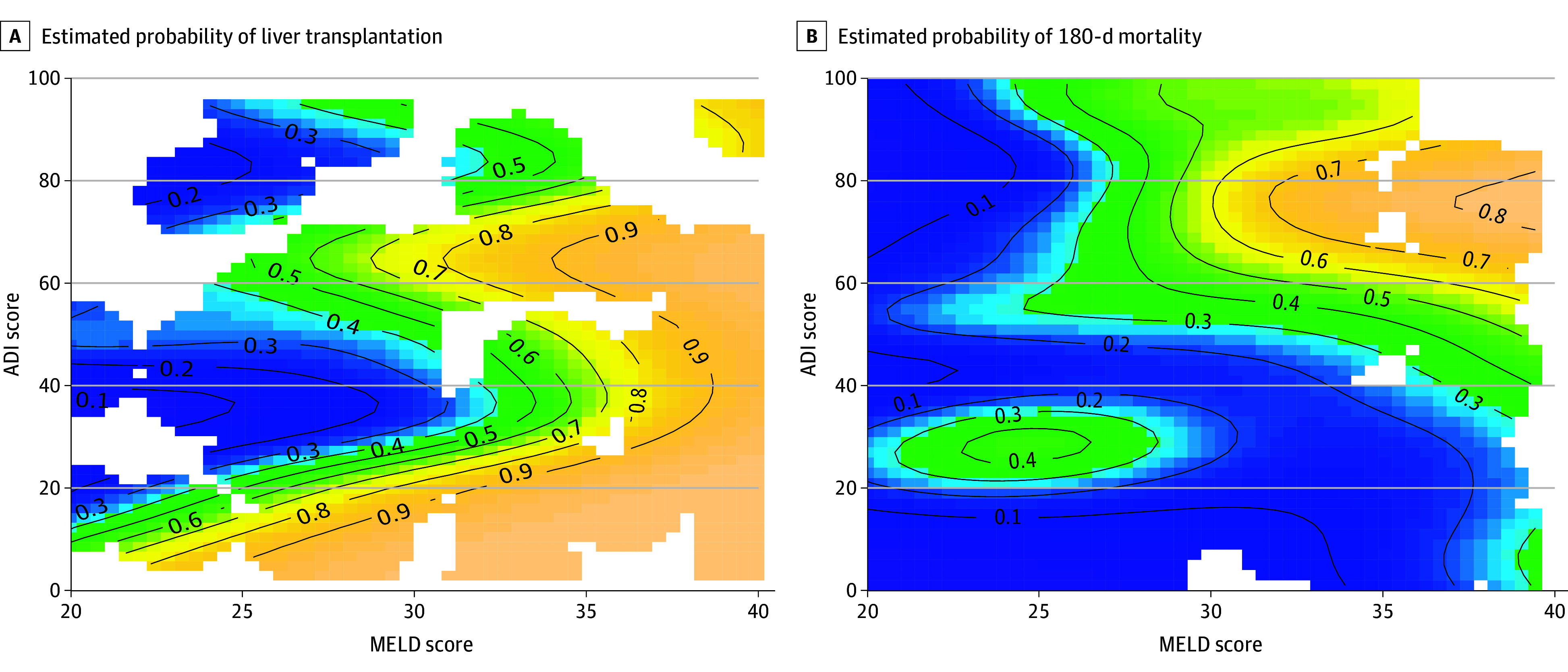
Association of the Area Deprivation Index (ADI) Score and the Model for End-Stage Liver Disease (MELD) Score With Liver Transplant and 180-Day Mortality Estimated probability of liver transplant (A) and 180-day mortality (B) as a smooth function of MELD and ADI scores. The MELD was the dominant factor associated with the likelihood of transplant, and mortality increased sharply with both MELD and ADI scores, with the steepest gradients for patients with high ADI and high MELD scores.

In the GAM model, the MELD × ADI interaction was not significant (*P* for interaction = .38). The estimated probability surface function showed that at MELD scores above 35, the probability of LT was uniformly high across the full ADI range, suggesting that clinical severity may outweigh social disadvantage at this stage of the transplant cascade (Figure 2A).

### Factors Associated With 180-Day Mortality

A total of 83 patients in the total cohort (25.5%) died within 180 days. In multivariable analysis, older age (OR, 1.03 [95% CI, 1.00-1.06]; *P* = .03) and higher MELD score (OR, 1.07 [95% CI, 1.03-1.12]; *P* < .001) were associated with mortality. ADI was not associated with increased mortality (OR, 1.01 [95% CI, 1.00-1.02]; *P* = .18) (Table 2).

In the GAM analysis for 180-day mortality, the MELD × ADI interaction was statistically significant (*P *for interaction = .01) (Table 3). The estimated probability surface function showed that mortality varied by ADI. Patients with an ADI less than 20 had 180-day mortality probabilities of 10% to 20% across all MELD scores. Mortality probability increased with both MELD and ADI. Patients with a low ADI (<30) had relatively low estimated mortality (approximately 10% to 20%) across all MELD strata. Among patients with a higher ADI (≥60), estimated mortality increased steeply with MELD from approximately 20% at MELD scores 20 to 30 to over 70% at a MELD more than 40. These gradients indicate a potential compounding association of clinical severity and neighborhood deprivation on survival (Figure 2B).

## Discussion

With rising rates of AH in the US and growing evidence supporting early LT as a life-saving intervention, understanding barriers to this therapy and ensuring equitable access are critical. Despite increased use of LT for ALD, little is known about access at stages prior to wait-listing. In this prospective multicenter cohort of patients with sAH, we examined how both clinical severity and social disadvantage were associated with progression through the LT care cascade, starting at referral, and their joint association with 180-day mortality in this population that is acutely ill. To our knowledge, this is the first prospective multicenter cohort to find that social disadvantage and disease severity interacted and were associated with referral patterns in sAH.

As expected, the MELD score was associated with the transplant cascade. However, we also found that neighborhood-level disadvantage, measured using the ADI, was independently associated with lower odds of wait-listing and with the outcome of MELD on referral, wait-listing, and mortality. Specifically, at the referral step, ADI was associated with MELD scores between 20 and 30, a clinically important range, in which transplant decisions are less automatic. Prior work shows that even moderate AH is associated with significant short-term mortality.^19^ In addition, these findings begin to untangle the complex association between medical urgency and social risk factors. The ADI was not associated with transplant receipt among wait-listed patients, suggesting that allocation decisions were associated with clinical severity. However, among patients with MELD scores more than 40, 180-day mortality exceeded 70% for those in the most disadvantaged neighborhood, identifying a high-risk subgroup that also may require targeted interventions. Notably, 74.5% of patients in this cohort were alive at 180 days, a survival rate somewhat higher than that reported in many sAH populations. This likely reflects the prospective design, tertiary-center supportive care, and careful characterization of sAH within AlcHepNet. Accordingly, for a subset of patients, clinical improvement may have reduced the need for transplant evaluation, and some patients without referrals likely represented appropriate clinical decision-making rather than missed opportunities.

Our findings reinforce prior research demonstrating that the referral step in the LT care cascade represents a substantial access barrier. In a large cohort of veterans of over 34 000 patients with decompensated cirrhosis, only 4.5% were referred for LT, and fewer than 2% were transplanted.^20^ Similarly, in a multicenter safety-net study, just 28% of eligible patients with cirrhosis were referred, and referral was less likely for those with public insurance, Black race, or limited English proficiency.^21^ Our prospective study adds to the literature by reporting a 37% referral rate among hospitalized patients with sAH and extends this literature by showing that referral is not only associated with clinical severity but also with its interaction with area-level social disadvantage. Because our cohort was drawn from academic centers, in which referral pathways are relatively well established, it is plausible that referral rates in smaller or nontransplant hospitals are even lower, potentially magnifying the disparities observed in our study.

Most existing studies of early LT in sAH focus on outcomes among patients already selected for transplant. The ACCELERATE-AH (American Consortium of Early Liver Transplantation for Alcoholic Hepatitis) consortium reported excellent 3-year survival (84%) among patients transplanted without mandated abstinence.^11^ Yet, the cohort was predominantly White, male, and publicly insured, raising concerns about which patients are referred for this intervention.^22^ Our study contributes to this literature by capturing the full LT cascade and examining the association of both area-level and individual-level SDOH with transplant access.

Our results also align with growing literature on structural barriers to transplant. Mohamed et al^9^ showed that neighborhood poverty was associated with both failure to be wait-listed and death during LT evaluation, even after adjusting for individual insurance status. Strauss et al^23^ further demonstrated that neighborhood deprivation interacts with race, limiting wait-listing, particularly for Black patients from high ADI areas. Building on these findings, our data revealed that the association with ADI was not static but shifted across levels of disease severity, supporting the need to consider both absolute risk and clinical context in equity-focused interventions.

Notably, some anticipated covariates were not significant in our model. In contrast to prior studies, individual-level SDOH, such as insurance type and educational level, were not independently associated with referral or wait-listing after adjusting for MELD and ADI. This may reflect the study context; although our analysis used prospectively collected data, transplant decisions were still captured retrospectively. Moreover, the parent study’s prospective design may have introduced a Hawthorne effect, whereby clinicians may have altered their decision-making due to awareness of being observed, potentially attenuating the apparent association with individual SDOH.^24^ Additionally, we observed significant center-level variability in referral patterns, with patients at some sites having 4 to 5 times higher odds of referral (eg, site 5: OR, 5.62 [95% CI, 1.68-18.83]), suggesting that structural factors such as institutional policies, staffing, or physician familiarity with LT for sAH may influence access.

These findings underscore the need for multilevel interventions to address LT disparities. At the community level, targeted outreach and navigation may support patients at high risk, particularly those from disadvantaged neighborhoods. At the transplant center level, standardizing evaluation protocols, integrating SDOH into clinical workflows, and equity-informed multidisciplinary review may reduce disparities in listing. At the policy level, funding for navigation programs and inclusion of social risk metrics in program evaluation may promote fair and timely access to LT for sAH.

### Strengths and Limitations

This study has several notable strengths. To our knowledge, it is the first analysis of a prospective multicenter cohort to evaluate the full LT care cascade, including referral, wait-listing, transplant, and mortality, among patients with sAH. The prospective design enabled standardized data collection and a detailed assessment of multiple SDOH. The cohort was geographically and demographically diverse, with meaningful representation of women and Black and Hispanic or Latino patients and included only patients with well-characterized, clinically diagnosed sAH, enhancing diagnostic accuracy and generalizability.

This study also has limitations. Although clinical and social data were collected prospectively, transplant decisions occurred as part of routine care and were captured retrospectively, limiting insight into practitioner decision-making. We lacked key SDOH variables, particularly postdischarge social support and substance use, which may strongly influence transplant eligibility and follow-up. We were also unable to assess medical contraindications or comorbidity burden and therefore could not assess whether these factors may have mediated the associations observed. Enrollment occurred during the COVID-19 pandemic, which may have introduced unmeasured operational variability despite adjustment for site. Sample sizes were smaller at later steps of the transplant cascade, limiting power to detect more nuanced associations. As an observational study, causal inference is not possible. Additionally, substantial site-level variation suggests that unmeasured institutional or regional factors may also contribute to disparities.

## Conclusions

This multicenter cohort study of patients with severe alcohol-associated hepatitis found that neighborhood disadvantage was associated with referral at intermediate MELD scores. Social risk was most consequential early in the transplant cascade, particularly among patients with intermediate MELD scores, identifying referral as a critical leverage point for equity-focused intervention. Ensuring timely and fair access to evaluation for patients from disadvantaged communities will be essential, as early LT for sAH continues to expand and as equity efforts extend across solid organ transplant.

## Notes

Supplement 1. **eTable 1. **Demographic and Social Determinants of Health of Patients With Severe Acute Alcohol-Related Hepatitis at Each Step in the Liver Transplant Evaluation Cascade **eTable 2. **Sample Sizes for Selected MELD and Area Deprivation Index (ADI) Strata Used From the Generalized Additive Model (GAM) **eTable 3.** Multivariable Logistic Regression for the Referral Step With Insurance and Site Interaction **eTable 4. **Generalized Additive Models (GAMs) Evaluating MELD × Area Deprivation Index (ADI) Interactions at the Referral Step With 4 Insurance Categories

Supplement 2.
Data Sharing Statement
