# Supplementary material for: Neighborhood Disadvantage and Access to Liver Transplant Referral for Severe Alcohol-Associated Hepatitis
Source: JAMA Netw Open. 2026 Mar 19;9(3):e262567. doi: 10.1001/jamanetworkopen.2026.2567 (PMC13003376; doi:10.1001/jamanetworkopen.2026.2567)
Supplement: Supplement 1. — eTable 1. Demographic and Social Determinants of Health of Patients With Severe Acute Alcohol-Related Hepatitis at Each Step in the Liver Transplant Evaluation Cascade eTable 2. Sample Sizes for Selected MELD and Area Deprivation Index (ADI) Strata Used From the Generalized Additive Model (GAM) eTable 3. Multivariable Logistic Regression for the Referral Step With Insurance and Site Interaction eTable 4. Generalized Additive Models (GAMs) Evaluating MELD × Area Deprivation Index (ADI) Interactions at the Referral Step With 4 Insurance Categories [file jamanetwopen-e262567-s001.pdf]

Supplemental Online Content

Nephew LD, Cotter T, Singal A, et al. Neighborhood disadvantage and access to liver transplant referral for severe alcohol-associated hepatitis. *JAMA Netw Open*. 2026;9(3):e262567. doi:10.1001/jamanetworkopen.2026.2567

eTable 1. Demographic and Social Determinants of Health of Patients With Severe Acute Alcohol-Related Hepatitis at Each Step in the Liver Transplant Evaluation Cascade
eTable 2. Sample Sizes for Selected MELD and Area Deprivation Index (ADI) Strata Used From the Generalized Additive Model (GAM)
eTable 3. Multivariable Logistic Regression for the Referral Step With Insurance and Site Interaction
eTable 4. Generalized Additive Models (GAMs) Evaluating MELD × Area Deprivation Index (ADI) Interactions at the Referral Step With 4 Insurance Categories

This supplemental material has been provided by the authors to give readers additional information about their work.

© 2026 Nephew LD et al. *JAMA Network Open*.

eTable 1. Demographic and Social Determinants of Health of Patients With Severe Acute Alcohol-Related Hepatitis at Each Step in the Liver Transplant Evaluation Cascade

Variable	Overall (N=325)	Referral (N=120)	Waitlisting (N=69)	Transplant (N=52)	180-day Mortality (N=83)
**Age at enrollment**	44.8 ± 10.2	43.5 ± 9.7	43.4 ± 9.8	43.4 ± 9.9	46.5 ± 10.6
**Gender**					
**Female**	128 (39.4%)	45 (37.5%)	29 (42.0%)	19 (36.5%)	35 (42.2%)
**Male**	197 (60.6%)	75 (62.5%)	40 (58.0%)	33 (63.5%)	48 (57.8%)
**Race**					
**Black or African American**	30 (9.2%)	6 (5.0%)	1 (1.4%)		10 (12.0%)
**Other or unknown**	15 (4.6%)	6 (5.0%)	2 (2.9%)	1 (1.9%)	2 (2.4%)
**White**	280 (86.2%)	108 (90.0%)	66 (95.7%)	51 (98.1%)	71 (85.5%)
**Ethnicity**					
**Hispanic or Latino**	38 (12.0%)	7 (6.0%)	4 (5.8%)	4 (7.7%)	9 (11.5%)
**Non-Hispanic**	279 (88.0%)	110 (94.0%)	65 (94.2%)	48 (92.3%)	69 (88.5%)
**MELD Score**	29.2 ± 7.6	32.3 ± 7.7	33.8 ± 8.1	35.8 ± 8.2	31.8 ± 8.0
**Education**					
**High school or less**	145 (45.5%)	39 (32.8%)	20 (29.4%)	14 (27.5%)	41 (50.0%)
**Trade school/College/Graduate program**	174 (54.5%)	80 (67.2%)	48 (70.6%)	37 (72.5%)	41 (50.0%)
**Insurance**					
**Private**	106 (32.7%)	54 (45.4%)	32 (47.1%)	25 (49.0%)	23 (27.7%)
**Medicaid**	79 (24.4%)	35 (29.4%)	16 (23.5%)	10 (19.6%)	19 (22.9%)
**Medicare**	42 (13.0%)	17 (14.3%)	14 (20.6%)	11 (21.6%)	8 (9.6%)
**None**	79 (24.4%)	12 (10.1%)	5 (7.4%)	4 (7.8%)	26 (31.3%)
**Other**	18 (5.6%)	1 (0.8%)	1 (1.5%)	1 (2.0%)	7 (8.4%)
**Employment**					
**No**	195 (61.1%)	66 (55.0%)	35 (50.7%)	23 (44.2%)	59 (72.0%)
**Yes**	124 (38.9%)	54 (45.0%)	34 (49.3%)	29 (55.8%)	23 (28.0%)
**Area deprivation Index**	56.2 ± 24.4	49.1 ± 25.1	42.1 ± 23.8	40.2 ± 23.8	59.6 ± 22.9
**Marital Status**					
**Divorced, separated, widowed or single**	186 (57.6%)	63 (52.9%)	30 (44.1%)	21 (40.4%)	53 (64.6%)
**Married or living with significant other**	137 (42.4%)	56 (47.1%)	38 (55.9%)	31 (59.6%)	29 (35.4%)

© 2026 Nephew LD et al. *JAMA Network Open*.

eTable 2. Sample Sizes for Selected MELD and Area Deprivation Index (ADI) Strata Used From the Generalized Additive Model (GAM)

Step in Cascade	n	MELD Category	ADI Category
**Referral**	24	20–30	<30
	171	20–30	≥30
	104	>30	All
**Waitlisting**	11	20–30	<30
	25	>30	<40
	33	>30	≥40
**Mortality**	87	20–30	≥60
	12	>40	≥60
	44	All	<30

© 2026 Nephew LD et al. *JAMA Network Open*.

eTable 3. Multivariable Logistic Regression for the Referral Step With Insurance and Site Interaction

		Analysis of Maximum Likelihood Estimates			
**Parameter**	DF	Estimate	Standard Error	Wald Chi-Square	Pr > ChiSq
**Intercept**	1	-16.6950	173.2	0.0093	0.9232
**Age at enrollment**	1	0.00145	0.0190	0.0058	0.9392
**Gender (Male)**	1	0.3134	0.3371	0.8642	0.3526
**Black or African American**	1	-0.4558	0.6143	0.5505	0.4581
**Other or unknown**	1	1.3272	0.7388	3.2272	0.0724
**Hispanic or Latino**	1	-0.3963	0.6853	0.3344	0.5631
**Meld_cap**	1	0.1432	0.0284	25.4024	<.0001
**Albumin**	1	0.3045	0.2739	1.2356	0.2663
**Trade School/College/Graduate program**	1	0.6148	0.3572	2.9619	0.0852
**Private**	1	9.1860	173.2	0.0028	0.9577
**Medicaid**	1	12.3056	173.2	0.0050	0.9434
**Medicare**	1	0.6698	182.0	0.0000	0.9971
**Other**	1	0.9293	178.8	0.0000	0.9959
**pr_adi**	1	-0.0111	0.00741	2.2377	0.1347
**Married or living with significant other**	1	0.0444	0.3635	0.0149	0.9028
**Children (No)**	1	-0.0910	0.4492	0.0410	0.8395
**Children (Yes)**	1	0.5064	0.4160	1.4817	0.2235
**Employed**	1	0.6564	0.3531	3.4562	0.0630
**Site 2**	1	10.3732	173.2	0.0036	0.9522
**Site 3**	1	11.0527	55.8243	0.0392	0.8431
**Site 4**	1	9.2896	173.2	0.0029	0.9572
**Site 5**	1	9.3871	173.2	0.0029	0.9568
**Private*site 2**	1	-8.0448	173.2	0.0022	0.9630
**Private*site 3**	1	-9.7476	55.8328	0.0305	0.8614
**Private*site 4**	1	-7.2729	173.2	0.0018	0.9665
**Private*site 5**	1	-6.9670	173.2	0.0016	0.9679
**Medicaid*site 2**	1	-11.6940	173.2	0.0046	0.9462
**Medicaid*site 3**	1	-11.8099	55.8334	0.0447	0.8325
**Medicaid*site 4**	1	-19.7673	199.3	0.0098	0.9210
**Medicaid*site 5**	1	-10.4654	173.2	0.0037	0.9518
**Medicare*site 2**	1	-9.7470	200.5	0.0024	0.9612
**Medicare*site 3**	0	0	.	.	.
**Medicare*site 4**	1	1.3393	182.0	0.0001	0.9941
**Medicare*site 5**	1	1.0836	182.0	0.0000	0.9952
**Other*site 2**	0	0	.	.	.
**Other*site 3**	0	0	.	.	.
**Other*site 4**	1	9.2027	251.9	0.0013	0.9709
**Other*site 5**	0	0	.	.	.

© 2026 Nephew LD et al. *JAMA Network Open*.

eTable 4. Generalized Additive Models (GAMs) Evaluating MELD × Area Deprivation Index (ADI) Interactions at the Referral Step With 4 Insurance Categories

	OR with 95% CI	p
**Gender: Male**	1.19(0.57, 2.5)	0.648
**Race: Black or African American vs. White**	0.63(0.19, 2.09)	0.452
**Race: Other or unknown vs. White**	3.63(0.81, 16.39)	0.093
**Hispanic**	1.19(0.3, 4.81)	0.803
**Education: Trade School/College/Graduate program**	2.40(1.16, 4.97)	0.019
**Medicare vs. Private**	0.64(0.22, 1.92)	0.427
**Medicaid vs. Private**	1.35(0.53, 3.43)	0.532
**Other vs. Private**	0.17(0.01, 2.78)	0.215
**None vs. Private**	0.15(0.05, 0.40)	<.001
**Age**	0.99(0.96, 1.03)	0.775
**Married or living with significant other**	0.92(0.43, 1.95)	0.819
**Children living at home: No vs. I do not have children**	1.41(0.54, 3.69)	0.487
**Children living at home: Yes vs. I do not have children**	1.86(0.76, 4.56)	0.177
**Employed**	2.20(1.04, 4.68)	0.040
**Albumin**	1.48(0.85, 2.57)	0.166
**Site 2**	6.37(1.29, 31.49)	0.023
**Site 3**	3.74(0.84, 16.57)	0.083
**Site 4**	4.02(0.87, 18.47)	0.074
**Site 5**	6.28(1.59, 24.83)	0.009
**s(ADI, meld_cap)**	EDF: 21.89	0.010

© 2026 Nephew LD et al. *JAMA Network Open*.
