# Supplementary material for: Neighborhood Disadvantage and Access to Liver Transplant Referral for Severe Alcohol-Associated Hepatitis
Source: JAMA Netw Open. 2026 Mar 19;9(3):e262567. doi: 10.1001/jamanetworkopen.2026.2567 (PMC13003376; doi:10.1001/jamanetworkopen.2026.2567)
Supplement: Supplement 2. — Data Sharing Statement [file jamanetwopen-e262567-s002.pdf]

Data Sharing Statement

Nephew. Neighborhood Disadvantage and Access to Liver Transplant Referral for Severe Alcohol-Associated Hepatitis. *JAMA Netw Open*. Published March 19, 2026. doi:10.1001/jamanetworkopen.2026.2567

Data
Data available: No

Additional Information
Explanation for why data not available: Upon request and approval by consortium.
